# *Plantago campestris* (Plantaginaceae), a rare new species from southern Brazil, supported by phylogenomic and morphological evidence

**DOI:** 10.7717/peerj.11848

**Published:** 2021-08-25

**Authors:** Gustavo Hassemer, Elliot M. Gardner, Nina Rønsted

**Affiliations:** 1 Câmpus de Três Lagoas, Universidade Federal do Mato Grosso do Sul, Três Lagoas, Mato Grosso do Sul, Brazil; 2 National Tropical Botanical Garden, Kalãheo, HI, United States of America; 3 International Center for Tropical Botany, Florida International University, Miami, FL, United States of America

**Keywords:** Endangered species, High-throughput sequencing, Identification key, Plantagineae, *Plantago commersoniana*, Taxonomy

## Abstract

High-throughput sequencing, when combined with taxonomic expertise, is a powerful tool to refine and advance taxonomic classification, including at the species level. In the present work, a new species, *Plantago campestris*, is described out of the *P. commersoniana* species complex, based on phylogenomic and morphological evidence. The main morphological characters that distinguish the new species from *P. commersoniana* are the glabrous posterior sepals and the slightly broader leaves. The new species is known from only three localities, all in natural high-elevation grasslands in Paraná and Santa Catarina states, southern Brazil. According to the IUCN criteria new species should be assessed as Endangered (EN). We present field photographs of *P. campestris* and related species, and we provide an identification key to the species previously included within the circumscription of *P. commersoniana*.

## Introduction

*Plantago* L. (order Lamiales Bromhead, family Plantaginaceae Juss., tribe Plantagineae Dumort.) is a cosmopolitan genus comprising ca. 250 species, with most of this diversity concentrated in temperate and high-elevation tropical areas (Pilger, 1937; Rahn, 1978; Rahn, 1996; Hassemer, De Giovanni & Trevisan, 2016; Iwanycki et al., 2019). The genus is notable for its reduced, wind-pollinated floral morphology (Primack, 1978; Kuiper & Bos, 1992; Hassemer et al., 2020b), highly variable vegetative morphology (Rahn, 1974; Rahn, 1992), complex taxonomy and still not adequately understood evolutionary history (Rønsted et al., 2002; Hoggard et al., 2003; Meudt, 2011; Hassemer et al., 2019; Höpke, Mucina & Albach, 2019). The genus has a rich history of ethnopharmaceutical uses, being widely used to treat a number of conditions (Samuelsen, 2000; Weryszko-Chmielewska et al., 2012; Gonçalves & Romano, 2016).

The predominantly South American *Plantago* sect. *Virginica* Decne. & Steinh. ex Barnéoud is the most species rich group within *Plantago* subg. *Plantago*, itself the most species rich subgenus in the genus (Rahn, 1974; Hassemer et al., 2019). Within this section, perhaps the most challenging group of species is the *P. commersoniana* Decne. & Barnéoud alliance (Hassemer, 2019). This species alliance is distributed in Bolivia, Paraguay, Uruguay, northeastern Argentina, central Mexico and southern, eastern and central Brazil (Rahn, 1974; Hassemer, 2019), and is characterised mainly by trichome characters, which are very important for the classification of *Plantago* (Rahn, 1992). This group includes the following currently-accepted species: *P. berroi* Pilg., *P. bradei* Pilg., *P. commersoniana*, *P. floccosa* Decne., *P. guilleminiana* Decne., *P. hatschbachiana* Hassemer, *P. pyrophila* Villarroel & J.R.I.Wood, *P. rahniana* Hassemer & R.Trevis., *P. veadeirensis* Hassemer and *P. weddelliana* Decne. (Hassemer, 2019). All these species (or their morphological circumscriptions, in the case of species described posteriorly), except *P. berroi*, *P. floccosa*, *P. guilleminiana* and *P. weddelliana*, were treated under the name *P. commersoniana* in the most recent comprehensive taxonomic treatment of *Plantago* sect. *Virginica* (Rahn, 1974) (see the identification key below).

The *Plantago. commersoniana* alliance constitute a group of plants which is hypothesised to have thrived during colder and drier periods in South America, when grasslands covered most of the land (Behling, 1998; Behling, 2002; Behling et al., 2007a). With the notable exceptions of *P. guilleminiana*, which occurs in high-elevation grasslands in southern Brazil, and *P. berroi*, which occurs in pampas in Uruguay and eastern Argentina, all members of this species complex are rare and threatened with extinction (Rahn, 1974; Hassemer, 2019), so that a good understanding of this group is therefore critical for biodiversity conservation (Ely et al., 2017; Thomson et al., 2018). It is illustrative that even the type-population of *P. commersoniana* was for a long time thought to be lost, having only been rediscovered two centuries after the collection of the type (Hassemer & Marchesi, 2016).

A number of new species were recently discovered and described out of the *Plantago. commersoniana* complex (Villarroel & Wood, 2011; Hassemer, Baumann & Trevisan, 2014; Hassemer, 2016; Hassemer, 2019), and one species (*P. bradei*, see Pilger, 1949) was re-established (Hassemer, 2017a). Phylogenetic reconstructions based on high-throughput sequencing, when combined with morphological data and taxonomic expertise, is a powerful tool to refine and advance taxonomic classification at the species level (*e.g.*, Gardner et al., 2016; Hou et al., 2016; Simpson et al., 2017; Uribe-Convers et al., 2017; Hassemer et al., 2019). In the present work, a new species is described out of the *P. commersoniana* species complex, based on a whole-chloroplast phylogenomic analysis and morphology. We present field photographs of the new species and related species, and we provide an identification key to the species previously (Rahn, 1974) included within the circumscription of *P. commersoniana*, which is still the most commonly used/seen name in herbaria worldwide for specimens of the *P. commersoniana* alliance.

## Materials & Methods

*Plantago* specimens kept at ASE, BHCB, C, CEN, CGMS, CIIDIR, DDMS, EAC, EFC, FI, FLOR, FT, FURB, GB, GH, HAS, HBR, HRB, HTL, HUFSJ, HURB, IAC, ICN, K, MA, MBM, MVFA, MVJB, MVM, P, PI, RB, SGO, TANG, TEPB, TUB, UB, UESC, UFMT, UPCB and UPS, and images of specimens kept at A, B, BBF, BM, BR, COI, CONC, CORD, CTES, DD, E, ESA, F, G, GJO, GOET, HFLA, IRAI, L, LD, LE, LINN, M, MO, MPU, MSNM, MW, PH, PRC, R, RO, ROV, S, SBT, SMDB, SP, TCD, UC, UEC, US, W and WU were studied (herbarium codes according to Thiers (2021) (continuously updated)). Specimens representative of the recorded geographic distributions of the species included in the *P. commersoniana* alliance were studied, comprising several hundred specimens in total. The classification of trichome types follows Rahn (1992). The diagnosis was prepared according to the recommendations in Hassemer, Prado & Baldini (2020a). The species concept adopted follows Hołyński (2005) and De Queiroz (2007). The taxonomic work followed the hypothesis-driven framework as described in Henderson (2005), Hołyński (2005) and Sluys (2013). The conservation status assessment follows the IUCN (2012), IUCN (2019) criteria. The distribution of the type specimens of the new species is pending the return of normalcy of herbaria activities, which is currently interrupted due to the COVID-19 pandemic.

To place the new species in a phylogenetic context, we generated phylogenetic trees based on whole chloroplast and nuclear ribosomal DNA (nrDNA) sequences. The chloroplast dataset consisted of 47 samples from the alignment used by Hassemer et al. (2019) (see table 2 in that work for voucher information), two additional genomes downloaded from GenBank (*P. lagopus* L. (accession no. MH205736, see Sun, Li & Wang, 2019) and *P. ovata* Forssk. (accession no. MH205737, see Li, Sun & Wang, 2019)), and new sequences generated for the new species. The nrDNA dataset was assembled from raw reads for the same samples.

For the sample of the new species, DNA was extracted from silica-dried leaf tissue of the type specimen (which serves as voucher) using the Qiagen DNeasy Plant Mini kit (Qiagen, Germany) following the manufacturer’s protocol and the modifications described in Hassemer et al. (2019). DNA was quantified using high sensitivity reagents on Qubit 2.0 fluorometer (Life Technologies, USA) and fragmented to ca. 300 bp with a Bioruptor (Diagenode, Belgium) for four cycles of 15 s ON / 90 s OFF. Illumina TruSeq-style libraries were prepared using the NEBNext DNA Ultra II kit following the manufacturer’s protocol. Libraries were amplified using AmpliTaq Gold (Life Technologies, USA) and quality checked on a TapeStation 2200 (Agilent Technologies, USA). Sequencing took place alongside other samples on a 2 ×  125 bp run on an Illumina HiSeq 2000.

To generate the chloroplast assembly, sequences were assembled de novo using NOVOPlasty 4.3.1 (Dierckxsens, Mardulyn & Smits, 2016). Because the sequences were not sufficient to assemble the entire plastome into a single contig in one run, we carried out two assemblies and combined them. The first assembly was seeded with a *P. major* L. *trnL–trnF* sequence extracted from the Hassemer et al. (2019) alignment, and the second was seeded with a *psbA* sequence from the same source. The three longest contigs from the two assemblies were aligned to *P. major* using MAFFT (Katoh & Standley, 2013) and manually combined using AliVew (Larsson, 2014) into a single scaffold with a gap of approximately 10kb. Sequences were aligned using MAFFT, columns with more than 75% gaps were removed with trimAl (Capella-Gutiérrez, Silla-Martínez & Gabaldón, 2009), and a maximum-likelihood tree was estimated under the best-fit model using IQtree (Minh, Nguyen & Haeseler, 2013). The figure was generated using ape 5.3 (Paradis, Claude & Strimmer, 2004) in R 3.5.1 (R Core Development Team, 2018). The plastome sequence of the sample of the type of the new species was deposited in GenBank (accession no. MW727694).

To generate the nrDNA sequences, raw reads were trimmed with Trimmomatic v.0.36 (ILLUMINACLIP: TruSeq3-PE.fa:2:30:10 HEADCROP:3 LEADING:30 TRAILING:25 SLIDINGWINDOW:4:25 MINLEN:20) (Bolger, Lohse & Usadel, 2014) and assembled with HybPiper, which produces gene-by-gene, reference-guided de novo assemblies (Johnson et al., 2016). The HybPiper reference consisted of *Plantago* sequences for ITS (GenBank accession no. AJ548971), 26S (KT179779), and 18S (KT179716), and the minimum coverage cutoff was set to 100x to ensure high-quality assemblies for this high-copy region. Sequences were aligned with MAFFT, visually inspected for any poorly-aligned regions, analysed with IQtree under the best-fit model. All analyses took place on a server hosted by Case Western Reserve University (USA), and the reads were deposited in the Sequence Read Archive (BioProject accession no. PRJNA729819).

The electronic version of this article in Portable Document Format (PDF) will represent a published work according to the International Code of Nomenclature for algae, fungi, and plants (ICN), and hence the new names contained in the electronic version are effectively published under that Code from the electronic edition alone. In addition, new names contained in this work which have been issued with identifiers by IPNI will eventually be made available to the Global Names Index. The IPNI LSIDs can be resolved and the associated information viewed through any standard web browser by appending the LSID contained in this publication to the prefix “http://ipni.org”. The online version of this work is archived and available from the following digital repositories: PeerJ, PubMed Central, and CLOCKSS.

## Results

### *Plantago campestris* Hassemer, sp. nov.

**Figure 1:**
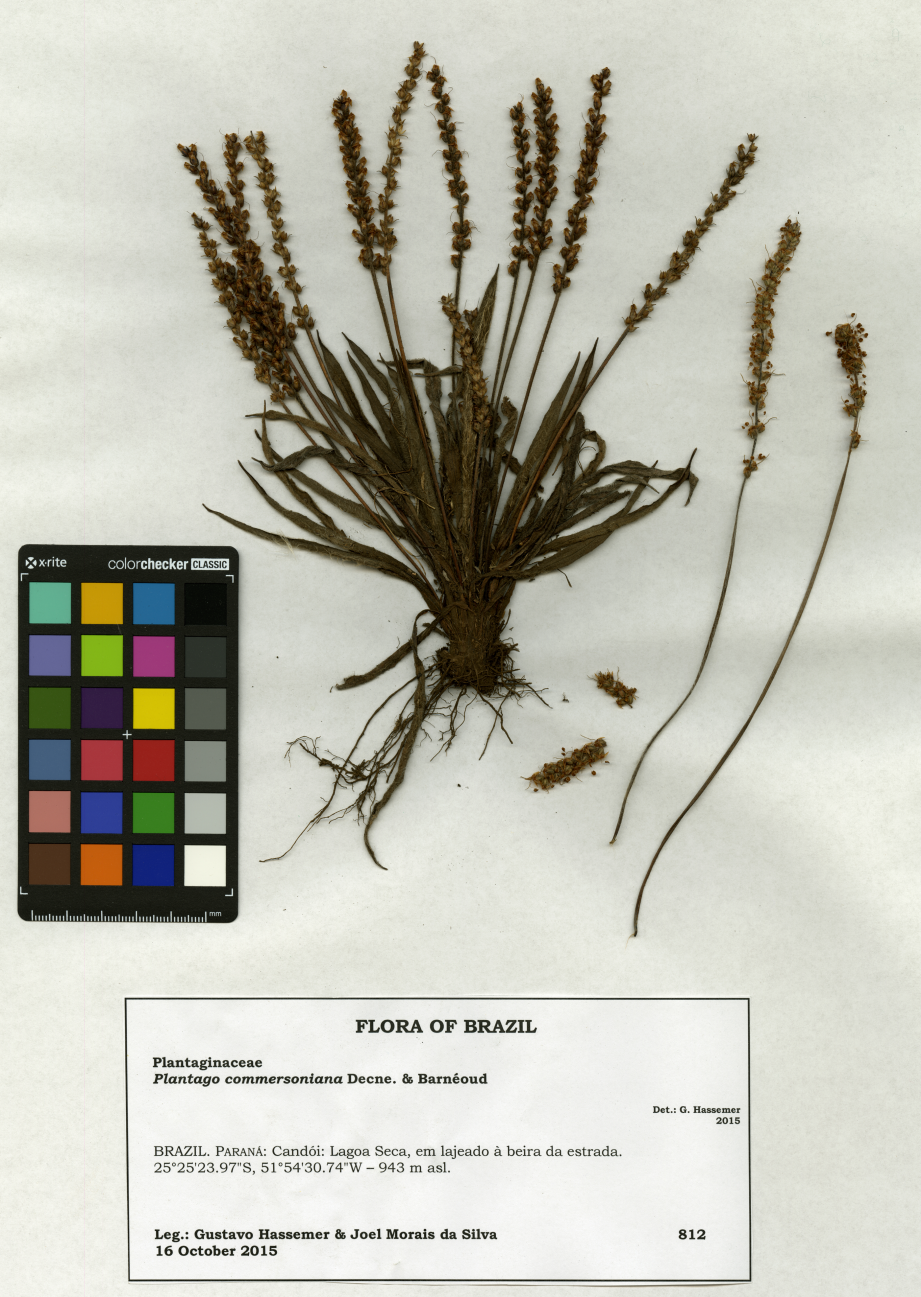
Image of the holotype of *Plantago campestris* (*G. Hassemer & J.M. da Silva 812*, HTL).

**Type:** BRAZIL. Paraná: Candói: Lagoa Seca, em lajeado à beira da estrada, 945 m, 16 October 2015, *G. Hassemer & J.M. da Silva 812* (holotype HTL! (Fig. 1); several isotypes to be distributed, to C, FT, FURB, HBR, MBM, OLD, among other herbaria).

**Diagnosis:** Similar to *Plantago. commersoniana*, from which it differs by its darkening more noticeably on drying, the slightly broader narrow-lanceolate leaves, and the glabrous sepals.

**Description:** Single-rosette herbs, up to 26 cm tall, perennial. Taproot developed, up to 5 cm long (probably much longer; it was broken in all specimens examined) and up to 0.9 cm wide; numerous unthickened (up to 1.5 mm wide) cord-like secondary roots also present. Caudex with negligible longitudinal growth, up to 1.7 cm wide, without a conspicuous crown of trichomes at its apex. Trichomes on leaves and scapes filiform, terete, with inconspicuous cellular articulations, very slender throughout their entire length, not perceptibly gradually tapering towards the apex (type K), whitish to light orange-coloured. Leaves 5.0–11.5 × 0.4–0.5 cm, papiraceous, 3-veined, basally attenuated, petiole indistinct from the narrow-lanceolate blade; apex acuminate; margin slightly to strongly involute, very sparsely microdenticulate (almost inconspicuously so); abaxial face covered with densely distributed, long (up to 12 mm long) trichomes giving a silky appearance; adaxial face with rather sparsely-distributed, shorter (up to three mm long) trichomes. Plant darkening appreciably on drying. Inflorescences 8.0–26.0 cm long. Scape 4.3–16.0 cm long, cylindrical, with evident longitudinal grooves, densely pilose throughout its extension, trichomes up to four mm long, variously-directed. Spike 3.7–11.0 cm long, (35–)50–80(–90)% of the length of the scape, cylindrical, multi-flowered, flowers less densely distributed in the lower part of the spike. Bracts linear-triangular, 2.1–2.4 × 0.5–0.6 mm, keeled; apex acuminate; glabrous except for relatively long (up to two mm long) very sparsely-distributed trichomes on the keel (dorsal face) and along the margins. Anterior sepals elliptic, 2.6–2.8 × 1.1–1.2 mm, keeled, glabrous; apex acute. Posterior sepals ovate, 2.7–2.8 × 1.3–1.5 mm, keeled, glabrous; apex acute. Corolla actinomorphic, glabrous, persistent after fruit maturation; lobes patent, 2.5–2.9 × 0.9–1.1 mm, elliptic, apex acuminate. Stamens 4; anthers 1.2–1.9 × 1.1–1.4 mm, purple, except whitish in the centre. Ovary with 2 ovules. Pyxidia 4.8–5.1 × 2.3–2.4 mm (including the persistent corolla), 2-seeded, with the corolla persisting after fruit maturation, fused to it. Seeds 2.4–2.7 × 1.1–1.3 mm, brown to blackish, ellipsoid, surface reticulate, convex on dorsal face, concave on ventral face.

**Figure 2:**
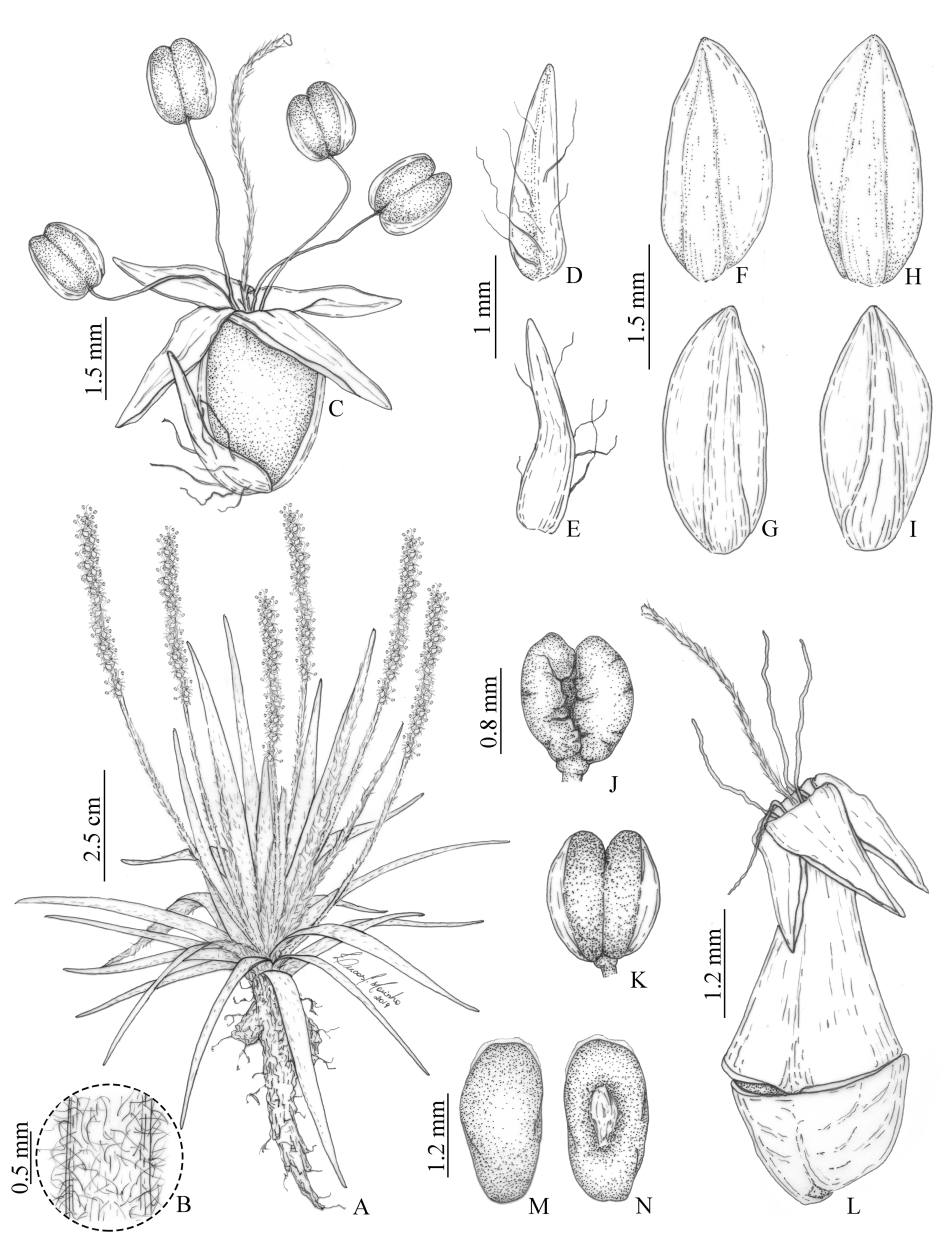
Illustrations of *Plantago campestris* based on the type gathering (*G. Hassemer & J.M. da Silva 812*). (A) Overview of specimen. (B) Detail of trichomes on scapes. (C) Flower. (D) Bract, dorsal face. (E) Bract, ventral face. (F) Anterior sepal, dorsal face. (G) Anterior sepal, ventral face. (H) Posterior sepal, dorsal face. (I) Posterior sepal, ventral face. (J) Anther, dorsal face. (K) Anther, ventral face. (L) Fruit (pyxidium). (M) Seed, dorsal face. (N) Seed, ventral face. Illustrations by L.C. Marinho.

**Illustrations:**Fig. 2.

**Figure 3:**
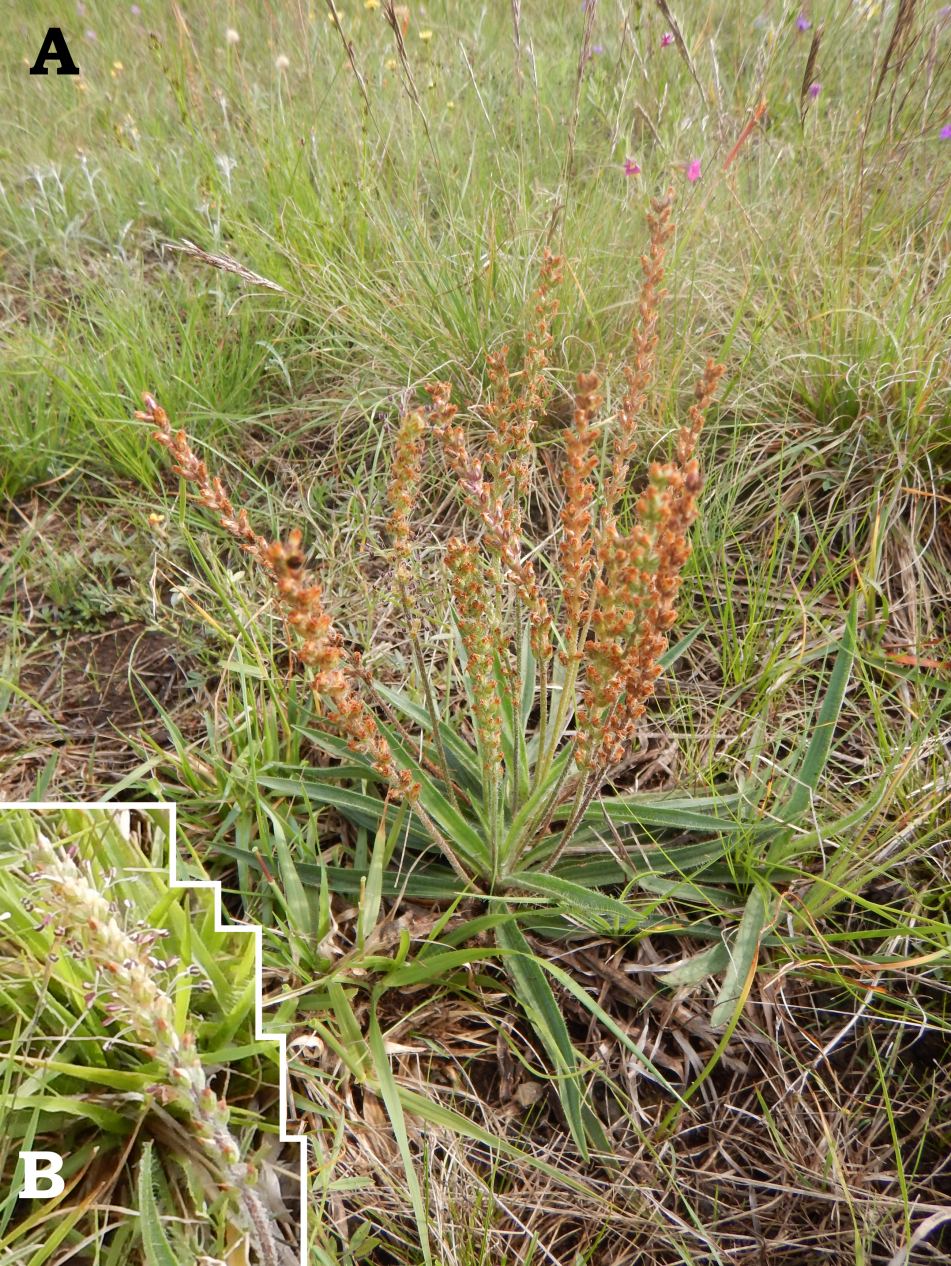
Field photographs of *Plantago campestris*. (A) Overview of specimen. (B) Detail of stamens.

**Photographs:**Fig. 1 (herbarium specimen); Fig. 3 (living specimen).

**Etymology:** The epithet is a reference to the habitat of the species, *i.e.,* the high-elevation grasslands of southern Brazil, which is perhaps the most neglected type of terrestrial environment in Brazil (Behling et al., 2007b; Overbeck et al., 2007; Overbeck et al., 2015), despite harbouring an impressive plant biodiversity and endemism (Iganci et al., 2011; Hassemer, Ferreira & Trevisan, 2015; Pla et al., 2020), frequently even greater than species-rich tropical forest areas. With over 35,683 plant species recorded in its territory, Brazil harbours the greatest plant biodiversity in the world (The Brazil Flora Group, 2015; The Brazil Flora Group, 2018; The Brazil Flora Group, 2021). However, conservation attention and efforts in the country have almost always focused on forests, which has often led to poorly-informed conservation decisions, favouring forest advance (which involves common, pioneer tree species) over the conservation of grasslands rich in endemic species. We hope that the description of narrowly-endemic new species from the Brazilian grasslands will help to draw attention to the importance of conserving these environments, promoting better-informed conservation decisions that take these unique ecosystems into account.

**Figure 4:**
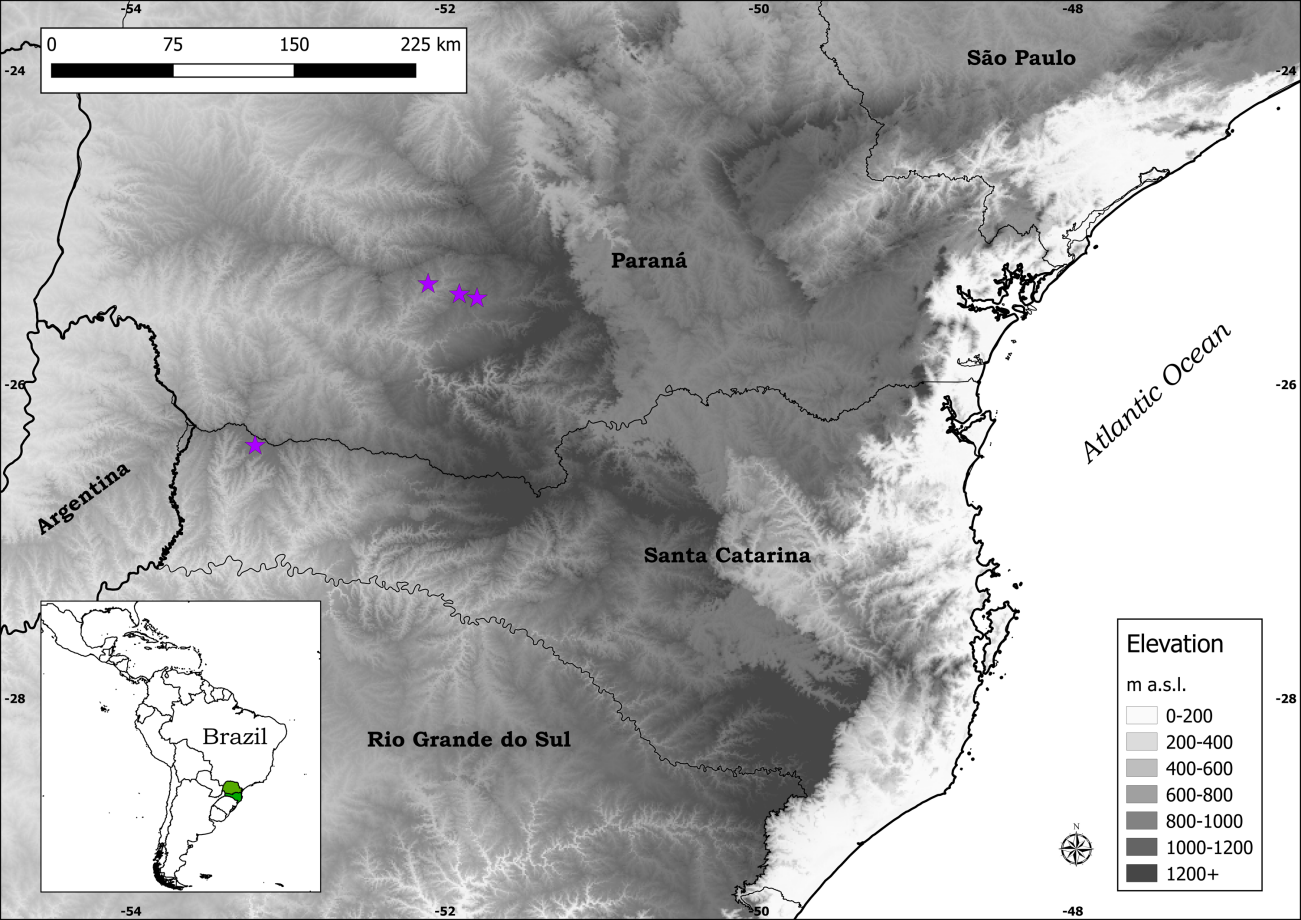
Distribution map of *Plantago campestris* (purple stars).

**Distribution:***Plantago campestris* is recorded in three municipalities in southern Brazil: Candói and Cantagalo, in the central-southern part of the state of Paraná, and Campo Erê, in the western part of the state of Santa Catarina (Fig. 4). It is important to mention that the municipalities of Candói and Cantagalo were both until recently part of the municipality of Guarapuava, hence the indication of the latter in labels of herbarium specimens.

**Figure 5:**
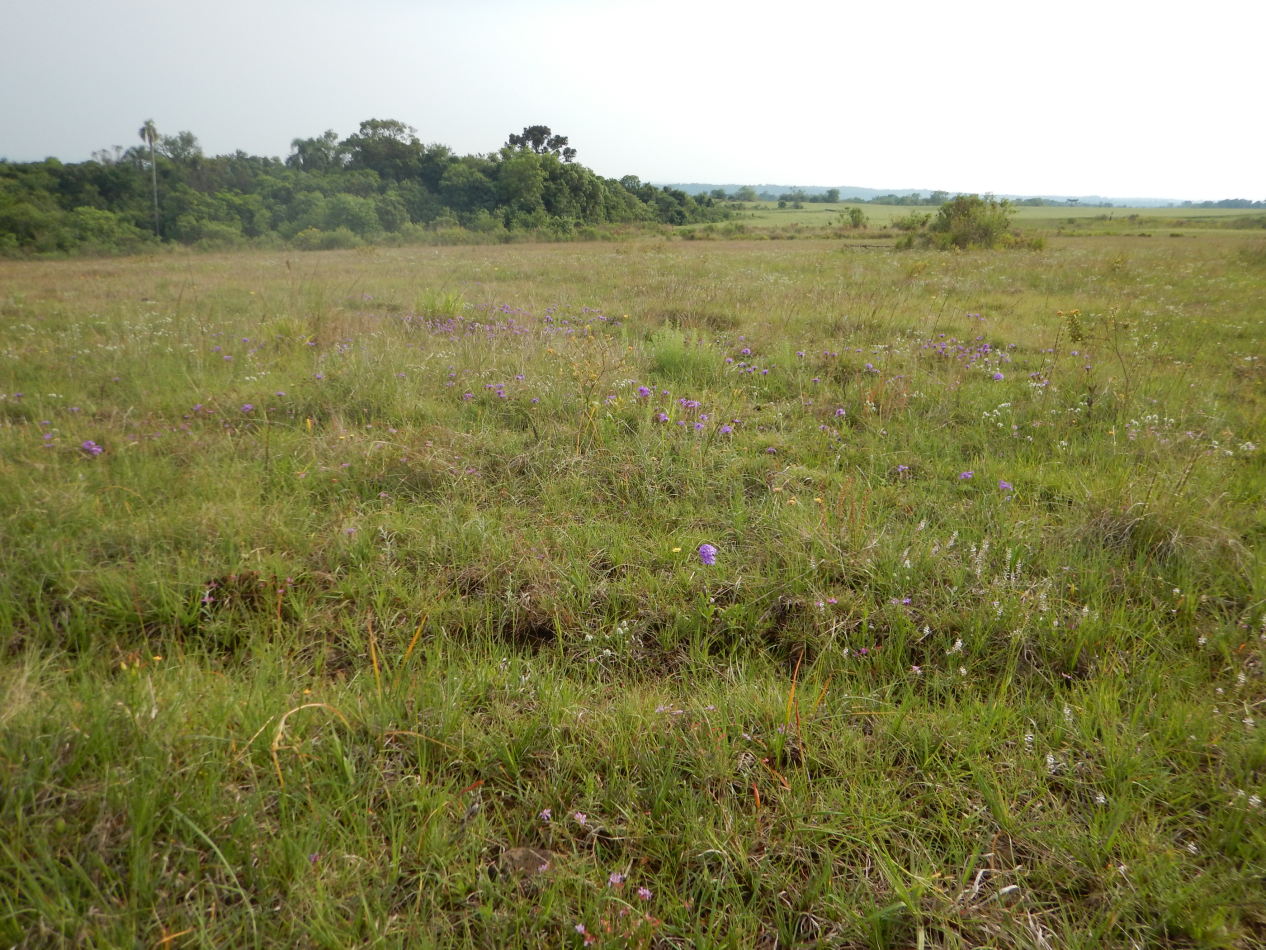
Habitat of the type-population of *Plantago campestris*, in the municipality of Candói, state of Paraná, southern Brazil.

**Habitat:** High-elevation grasslands (Fig. 5; see also comments and references above, under Etymology), at elevations of 750–1,000 m asl.

**Conservation status:** Endangered: EN—B2 (a, b[ii, iii, iv]). Only four populations are known, encompassing a total area of occupancy of <2 km^2^. As mentioned above, the high-elevation grasslands are a considerably neglected and highly threatened type of environment, with a clear ongoing tendency of area and quality reduction (Behling et al., 2007b; Overbeck et al., 2007; Overbeck et al., 2015; Hassemer, Ferreira & Trevisan, 2015). The main threats to the high-elevation grasslands in the region are the agricultural advance, and to a lesser extent the urban advance and the forest advance (due to the removal of cattle, see *e.g.*, Boldrini & Eggers, 1996; Sühs, Giehl & Peroni, 2020). Furthermore, none of the four recorded populations are within an environment protection area. Finally, the date of the most recent gathering is considerably old (>40 years) for all but one population, despite the fact that the states of Paraná and Santa Catarina can be considered well-sampled and are target of appreciable ongoing botanic sampling. This means that it is unfortunately possible that some of the recorded populations might be no longer extant. The first author, together with J.M. da Silva searched for the species in 2015 in suitable environments in adjacent areas in the state of Paraná but could not locate any further populations.

**Chromosome number:** unknown.

**Figure 6:**
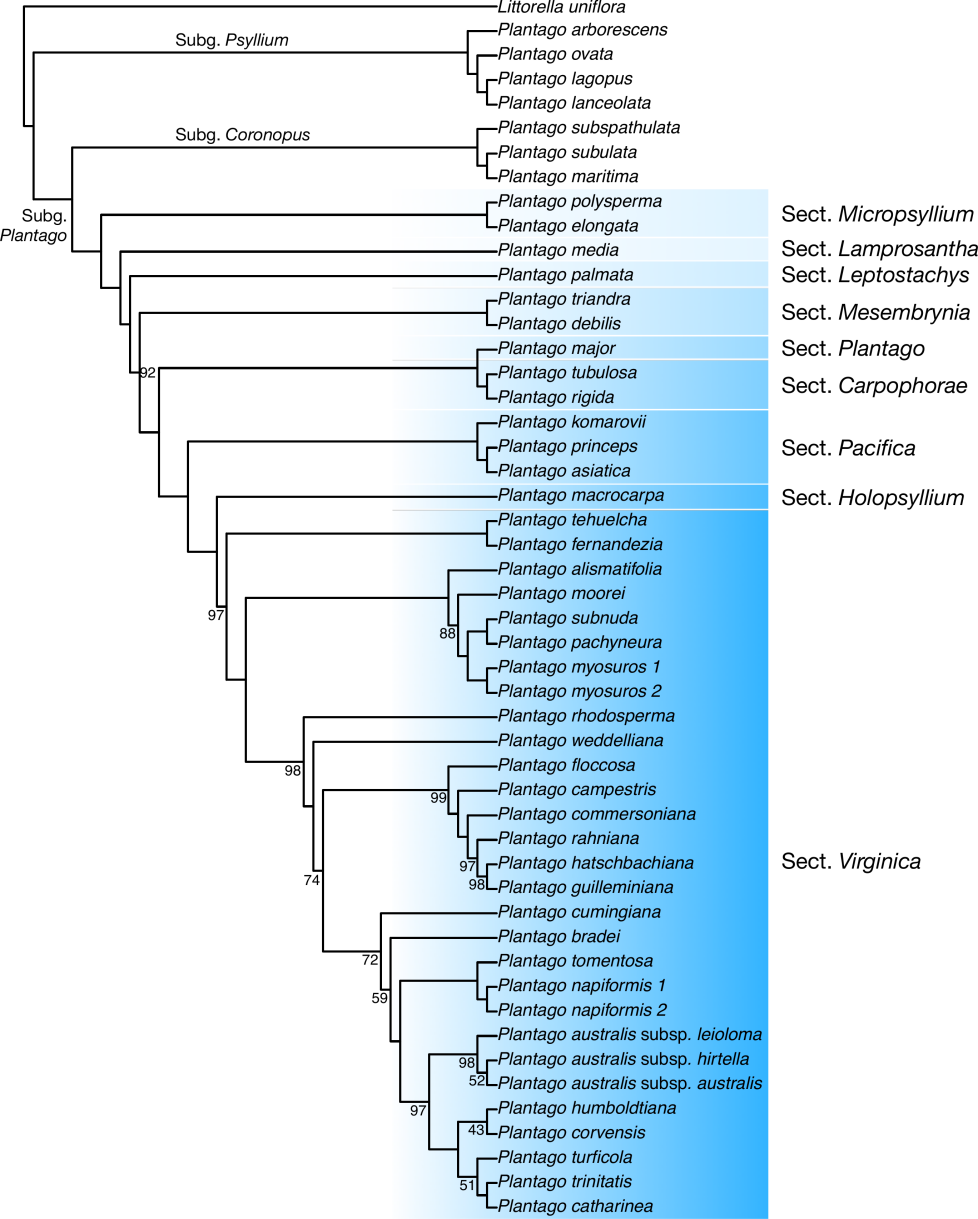
Maximum-likelihood tree based on whole chloroplast genomes showing the position of *Plantago campestris*. Bootstrap support values other than 100% are indicated.

**Figure 7:**
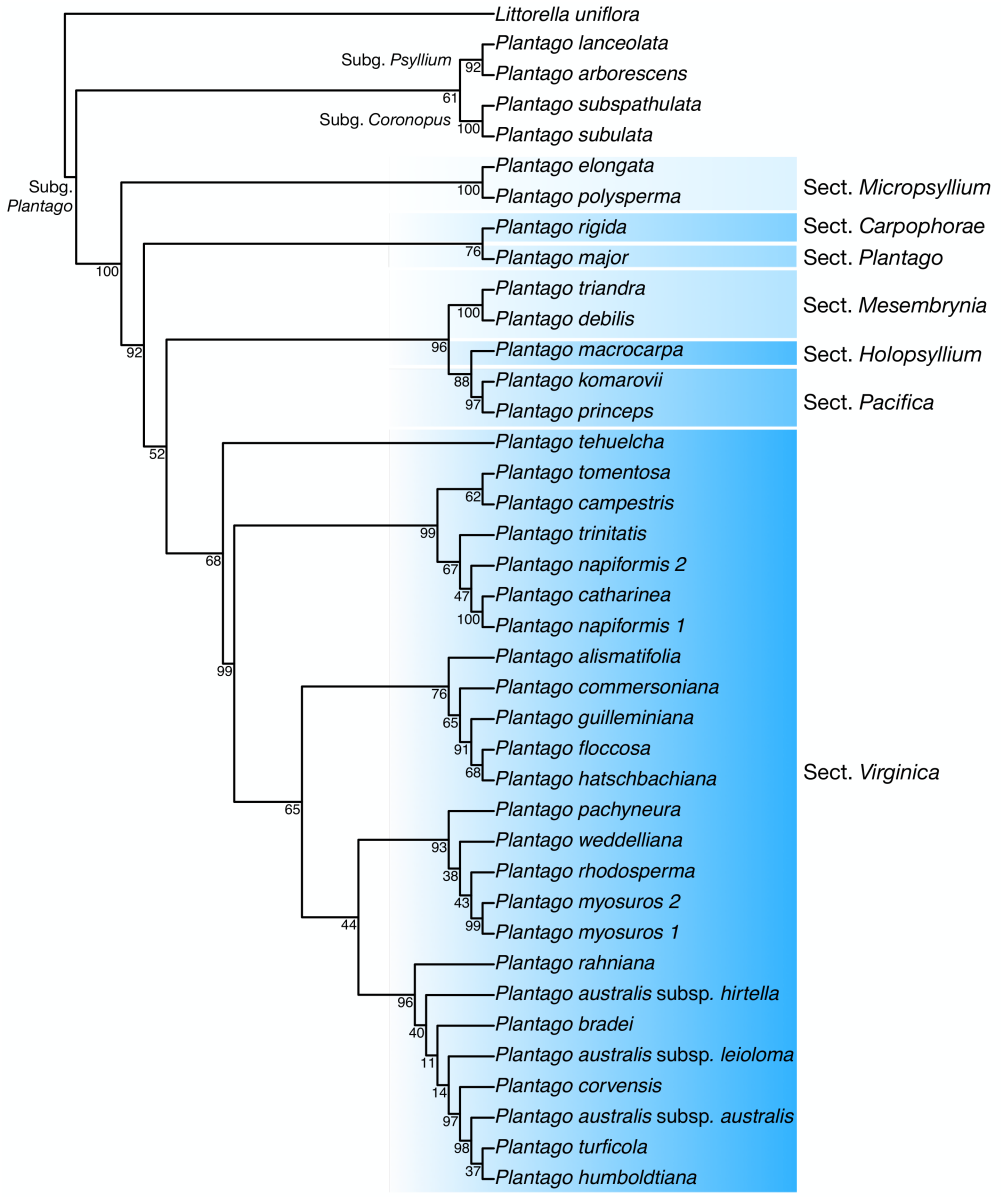
Maximum-likelihood tree based on nuclear ribosomal DNA sequences showing the position of *Plantago campestris*.

**Discussion:** In our plastome phylogenetic analysis (GTR+F+R5 model, log-lik =-334825.9250, AICc =669878.0542) (Fig. 6), the species in the *Plantago. commersoniana* alliance included in this study (*i.e., Plantago. campestris*, *P. commersoniana*, *P. floccosa*, *P. guilleminiana*, *P. hatschbachiana* and *P. rahniana*) formed a clade (BP = 100%) that does not include *P. bradei* and *P. weddelliana*. Within this *P. commersoniana* alliance clade, *P. guilleminiana* is sister to *P. hatschbachiana* (BS = 98%), followed by a grade of *P. rahniana* (BP = 97%), *P. commersoniana* (BP = 100%), *P. campestris* (BP = 100%) and *P. floccosa* (BP = 99%). Of the species in the *P. commersoniana* alliance, only *P. berroi*, *P. pyrophila* and *P. veadeirensis* were not sampled in this study. Based on morphology, we expect that *P. hatschbachiana* (sampled in this study), *P. pyrophila* and *P. veadeirensis* would form a clade. We cannot predict the position of *P. berroi* except that it most likely belongs to the clade of the *P. commersoniana* alliance. The remainder of the topology was identical to that found in Hassemer et al. (2019). The nrDNA tree (TVM+F+R2 model, log-lik =-12851.7151, AICc =25873.8488) was not as well resolved as the plastome tree, with only 13 nodes receiving at least 80% bootstrap support (Fig. 7). Thus, although the sectional clades agree with those in the plastome tree, the positions of individual species within sect. *Virginica* are often at odds with it. In the nrDNA tree, *P. campestris* is not part of the *P. commersoniana* clade, instead appearing in a clade with *P. catharinea*, *P. napiformis*, *P. trinitatis* and *P. tomentosa*.

**Figure 8:**
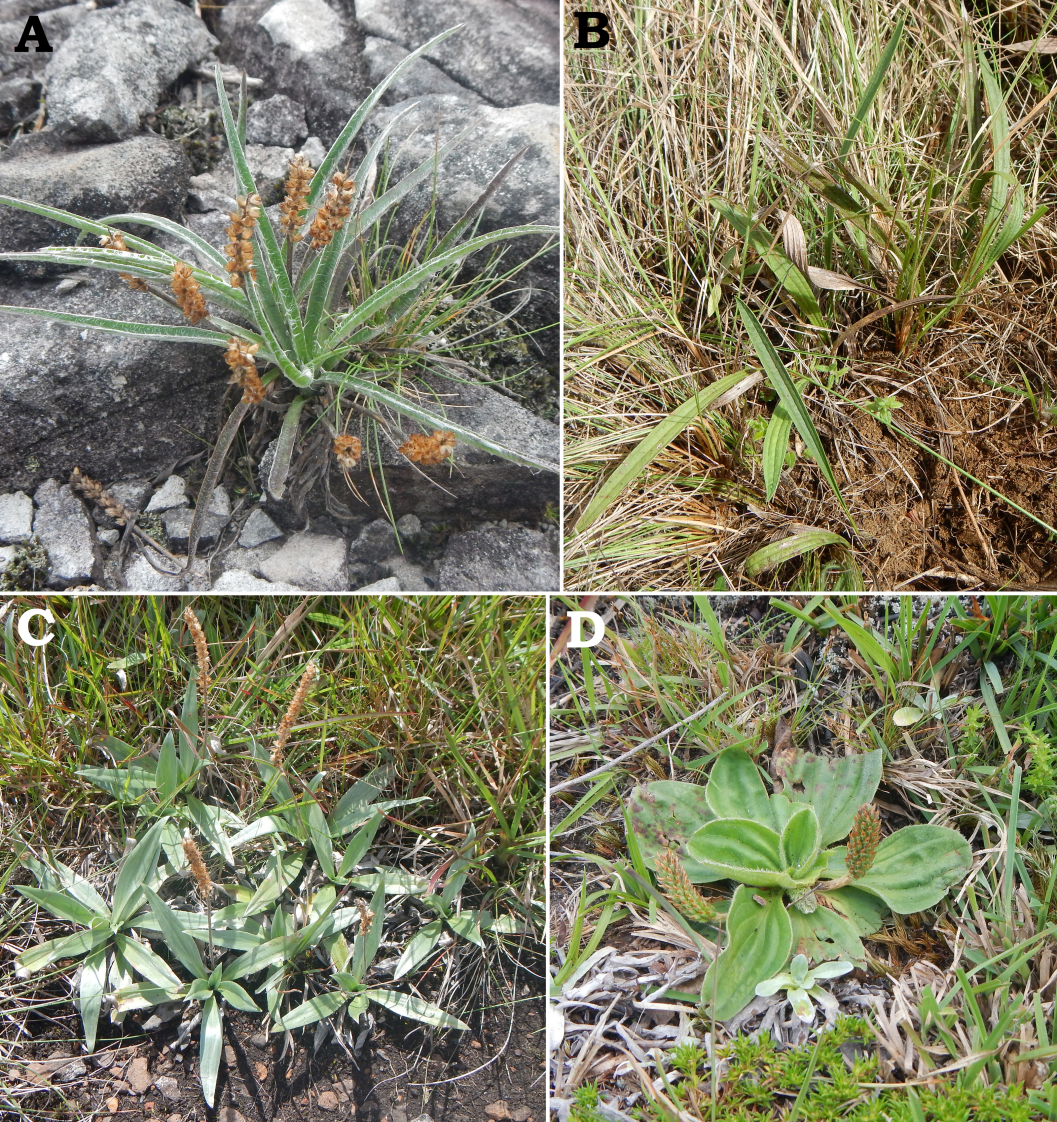
Field photographs of *Plantago commersoniana* (A), *P.  hatschbachiana* (B), *P. rahniana* (C) and *P. guilleminiana* (D). Photographs by G. Hassemer.

The position of the new species as sister to a clade including *P. commersoniana*, *P. guilleminiana*, *P. hatschbachiana* and *P. rahniana*, with very high support values throughout, indicates that the circumscription of *P. commersoniana* prior to the description of *P. campestris* was paraphyletic. In the nrDNA tree, the latter two species do not even form a grade. Furthermore, the striking morphological (Fig. 8), ecological and geographic (Hassemer, 2017b; Hassemer, 2019) differences between the four species that constitute the clade sister to *P. campestris* provide a compelling argument for the recognition of *P. campestris* at the species rank. This molecular phylogenetic result confirmed long-time taxonomic suspicions by the first author, despite the subtle morphological differences between *P. campestris* and *P. commersoniana* (see the diagnosis above). See Fig. 3 in Hassemer (2019) for the recorded distributions of *P. bradei*, *P. hatschbachiana*, *P. pyrophila*, *P. rahniana* and *P. veadeirensis*, Fig. 7 in Hassemer (2017b) for the distribution of *P. commersoniana*, and Fig. 55 in Rahn (1974) and Fig. 2A in Hassemer, De Giovanni & Trevisan (2016) for the distribution of *P. guilleminiana*.

**Additional specimens studied (paratypes):** BRAZIL. Paraná: Municipality of Candói: Rio Campo Real, 21 October 1966, *J. Lindeman & H. Haas 2769* (MBM-9311); Lagoa Seca, campo pedregoso e úmido, 21 September 1968, *G.G. Hatschbach 19777* (C, MBM-8773, UPCB-10161); Rio Campo Real, campo pedregoso, 1 October 1980, *G.G. Hatschbach 43207* (MBM-67967); Municipality of Cantagalo: campo pedregoso, 7 February 1969, *G.G. Hatschbach 21037* (C, MBM-16367); Santa Catarina: Municipality of Campo Erê: 6–24 km west of Campo Erê, rocky barren, 900–1,000 m, 20 February 1957, *L.B. Smith & R.M. Klein 11544* (HBR-31534).

### Identification key to the species encompassed within Rahn’s (1974) circumscription of *Plantago commersoniana*

Species distributions are presented within brackets.

**Table:** 

1. Leaves with a dense cover of silvery trichomes on both faces .............................................................................................................2
1′. Leaves without a dense cover of silvery trichomes on both faces .......................................................................................................3
2. Caudex generally inconspicuous, never growing horizontally. Leaves coriaceous. Pyxidia 3-seeded [Serra do Caparaó, eastern Brazil]......................................................................................... .........................................................................................*Plantago bradei*
2′. Caudex elongated, growing horizontally. Leaves chartaceous. Pyxidia 1–2-seeded [southern Santa Catarina, southern Brazil]........................................................... ................................................................................................................. *Plantago rahniana*
3. Leaves with the abaxial face densely covered with silvery/whitish trichomes, giving a silky appearance..........................................4
3′. Leaves with the abaxial face glabrous to pilose, but never densely covered with silvery/whitish trichomes....................................5
4. Leaves narrow-lanceolate. All sepals glabrous [southern Paraná and western Santa Catarina, southern Brazil]............................................................................................................*Plantago campestris*
4′. Leaves linear. Posterior sepals pilose on the keel [Uruguay, Paraguay and southern Brazil] ............................................................................................................................................................................. *Plantago commersoniana*
5. Leaves membranaceous, glabrous (even when young). Seeds with the ventral side deeply concave [Chapada dos Veadeiros, central Brazil].................... ............................................................................................ *Plantago veadeirensis*
5′. Leaves coriaceous, pilose (but gradually losing trichomes as they senesce). Seeds with the ventral side plane to slightly concave................................................ ...................................................................................................................................................... 6
6. Thickened taproot present, tuberous roots absent. Trichomes on leaves and scapes with conspicuous dark cellular articulations. Pyxidia 1–2-seeded [eastern Paraná, southern Brazil] ..................................................................................... *Plantago hatschbachiana*
6′. Roots formed of several subcylindrical tubers. Trichomes on leaves and scapes without conspicuous dark cellular articulations. Pyxidia 1-seeded [eastern Bolivia] ............................................................................................................................... *Plantago pyrophila*

## Conclusions

This work advances the taxonomic classification of *Plantago* sect. *Virginica* and of the *P. commersoniana* alliance by evidencing a narrowly endemic, endangered new species by the combination of phylogenomic and morphological evidence. Nevertheless, many issues remain unsolved in the classification of this group of organisms, which will require further sampling and study for their resolution. In order to prevent further irreversible loss of biodiversity, more attention and funding should urgently be directed towards protecting the Brazilian high-elevation grasslands and the many species endemic to these notable environments.

## Supplemental Information

10.7717/peerj.11848/supp-1 Supplemental Information 1 Alignment

10.7717/peerj.11848/supp-2 Supplemental Information 2 nrDNA alignment

## Additional Information and Declarations

### Field Study Permissions

The following information was supplied relating to field study approvals (i.e., approving body and any reference numbers): The type specimens of the new species were collected by G. Hassemer and J.M. da Silva in 2015. The specimens were collected outside any conservation area. G. Hassemer was then a PhD student financed by the Brazilian government, and J.M. da Silva was then a public servant working at a Brazilian herbarium, i.e., MBM herbarium (he has since retired). In accordance with the Brazilian legislation, Brazilian citizens do not need a permit to collect plants if the species is not in the official Red List and if the plants are not in an environmental conservation area. Thus, no permit was required for the collection of the type gathering of the new species.

### DNA Deposition

The following information was supplied regarding the deposition of DNA sequences: The plastome sequence of the sample of the type of the new species is available in GenBank: MW727694. *Plantago* sequences for ITS (AJ548971), 26S (KT179779), and 18S (KT179716) are available at the Sequence Read Archive (BioProject PRJNA729819).

### Data Availability

The following information was supplied regarding data availability: The alignments used for our phylogenetic analyses are available in the Supplemental Information. Voucher information for all samples used (except that of the new species) is available in Table 2 of the following work: Hassemer, G., Bruun-Lund, S., Shipunov, A.B., Briggs, B.G., Meudt, H.M. & Rønsted, N. 2019. The application of high-throughput sequencing for taxonomy: the case of *Plantago* subg. Plantago (Plantaginaceae). Molecular Phylogenetics and Evolution 138: 156–173. https://doi.org/10.1016/j.ympev.2019.05.013
The voucher for the sample of the new species is the holotype of the species, which is deposited in the HTL herbarium.

### New Species Registration

The following information was supplied regarding the registration of a newly described species: *Plantago campestris* LSID: 77218209-1
